# Supplementary material for: Nutrient-dependent regulation of a stable intron modulates germline mitochondrial quality control
Source: Nat Commun. 2024 Feb 10;15:1252. doi: 10.1038/s41467-024-45651-y (PMC10858910; doi:10.1038/s41467-024-45651-y)
Supplement: Supplementary file 1 — Supplementary Information [file 41467_2024_45651_MOESM1_ESM.pdf]

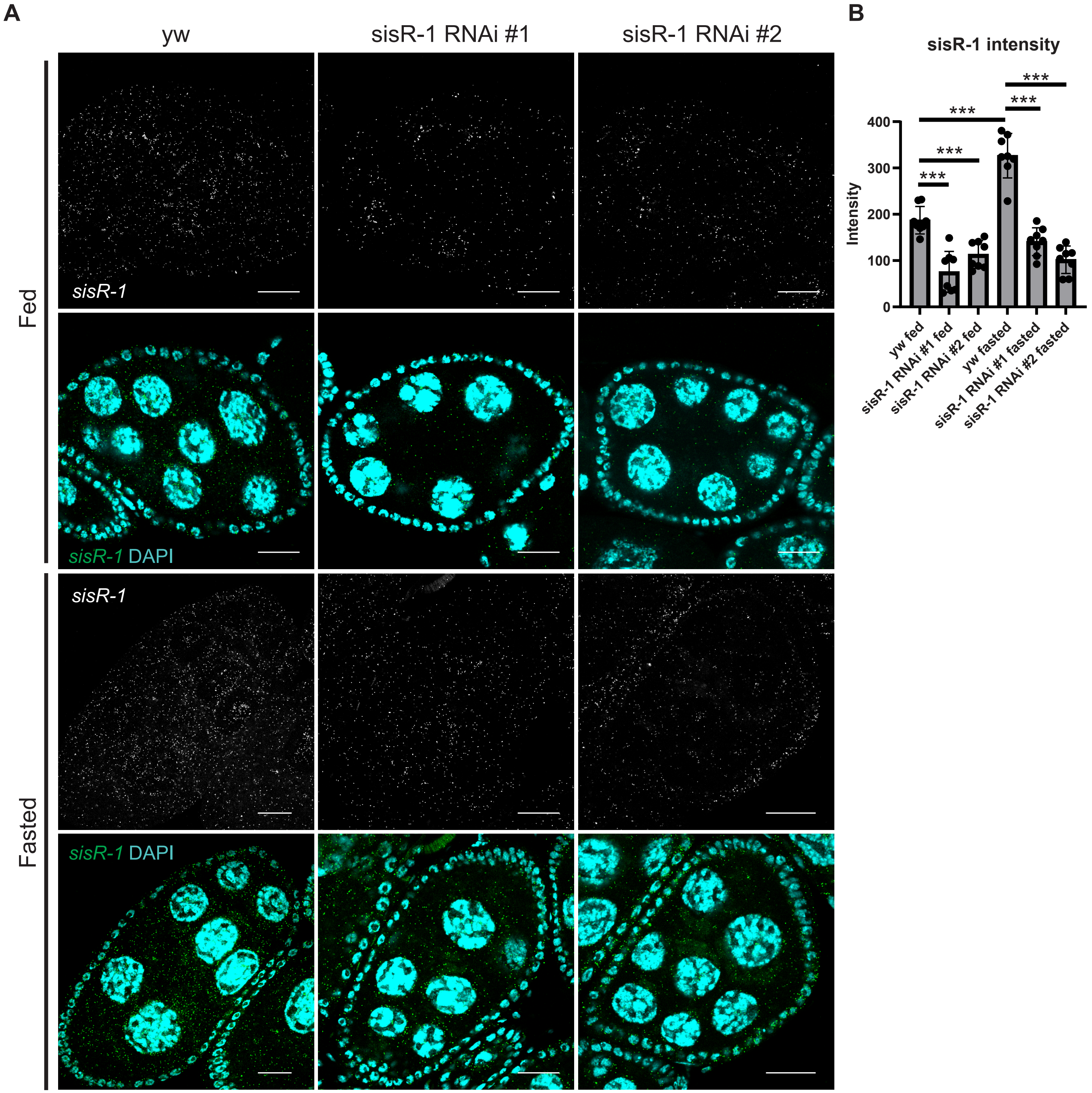

Fig S1. smFISH of sisR-1 in control and sisR-1 RNAi ovaries. (A) Confocal images of sisR-1 (Green) and DAPI (Blue) localizations in ovaries of fed and fasted Oregon R and sisR-1 RNAi. Scale bar = 20μm. (B) Relative expression of sisR-1 as measured by smFISH in the cytoplasm of stage 8/9 egg chambers in ovaries of fed and fasted Oregon R and sisR-1 RNAi. Data are presented as mean values +/- SD. ***: $p < 0.001$. n = 8 egg chambers counted per group. Two-tailed t-test.

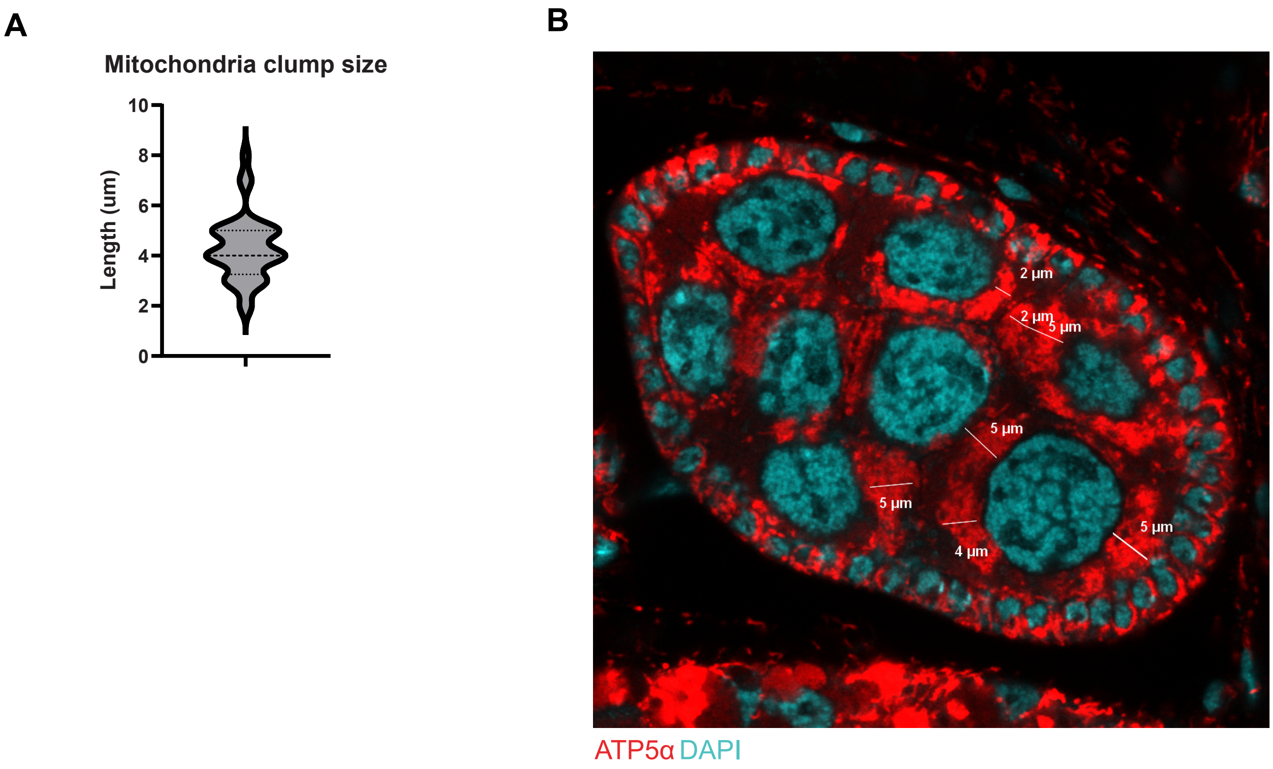

Fig S2. Characterization of mitochondrial clump size. (A) Violin plot showing the distribution of mitochondrial clump size. n = 6 clumps/egg chamber and 6 egg chambers. (B) Confocal image showing examples of the measurement of clump size. ATP5α (Red), DAPI (Blue).

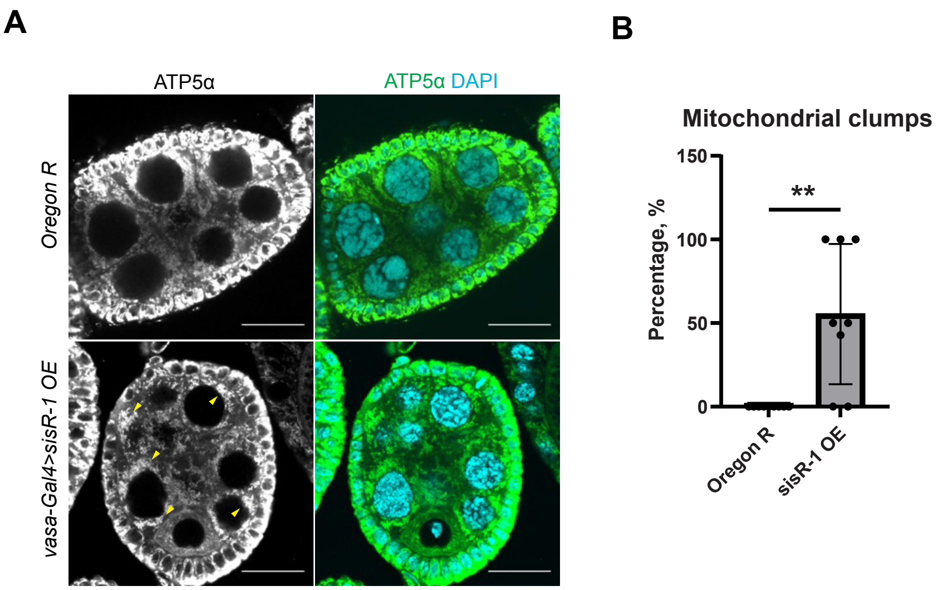

Fig S3. Overexpression of sisR-1 is sufficient to induce mitochondrial clumps. (A) Confocal images of Oregon R and vasa-Gal4>sisR-1 OE females fed with yeast paste for 2 days. ATP5α (Green) and DAPI (Blue). Arrowheads point to mitochondrial clumps. Scale bar = 20μm. (B) Chart showing percentages of nurse cell per stage 8/9 egg chamber with mitochondrial clumps in ovaries of Oregon R and vasa-Gal4>sisR-1 OE. Data are presented as mean values +/- SD. ***: $p < 0.001$. n = 9 (Oregon R), 8 (OE) egg chambers counted. Two-tailed t-test.

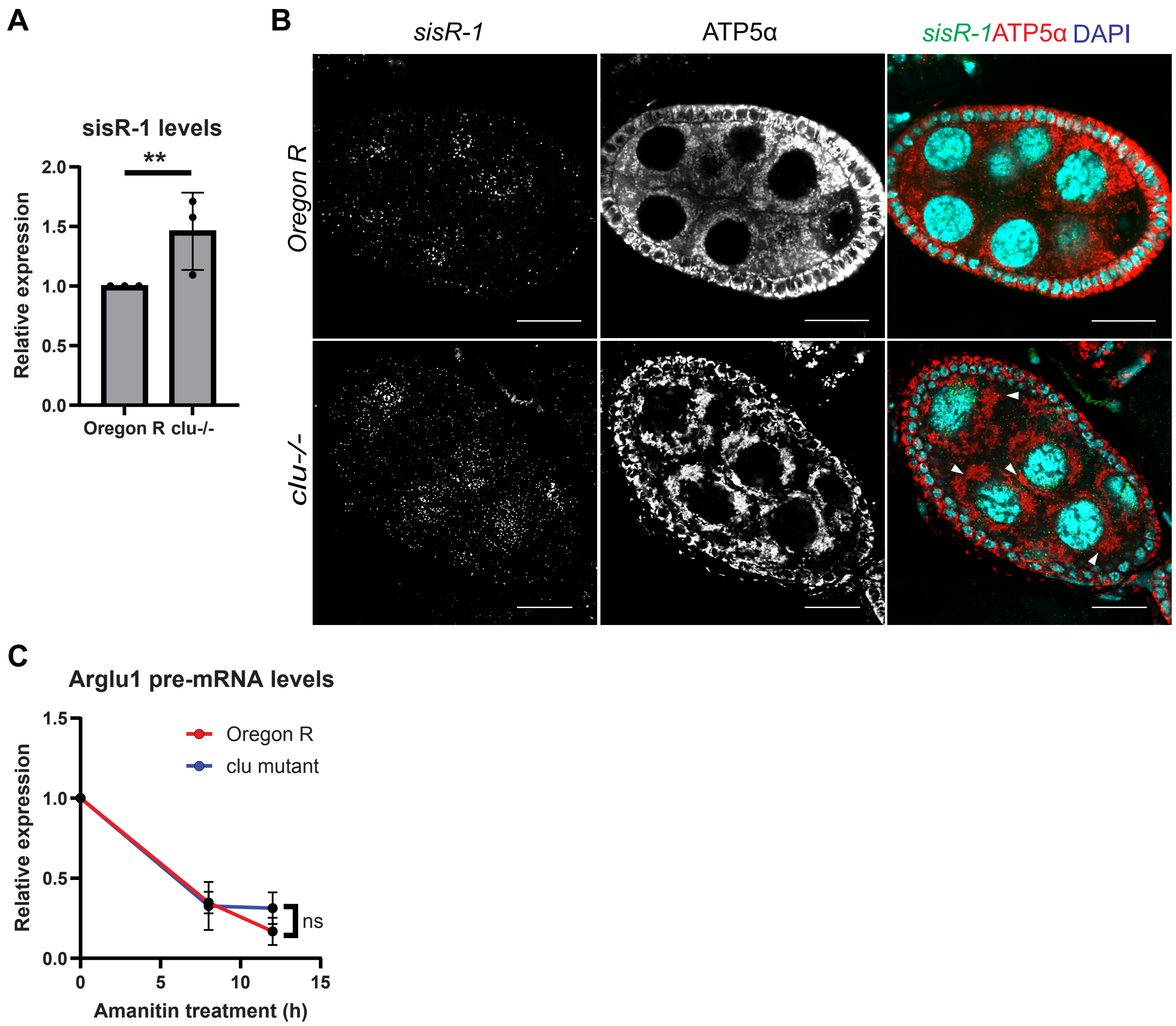

Fig S4. clueless regulates sisR-1 expression. (A) qRT-PCR showing the relative expression of sisR-1 expression in control Oregon R and clu[d08713] homozygous stage 14 oocytes. Data are presented as mean values +/- SD. from three biological replicates. *: p<0.05. Two-tailed t-test. (B) Confocal images of stage 8/9 egg chambers showing localization of sisR-1 (Green), ATP5α (Red) and DAPI (Blue) in fed Oregon R and fed clu[d08713] homozygous females. Arrowheads point to colocalization of sisR-1 and mitochondrial clumps in clu[d08713] homozygous females. Scale bar = 20μm. (C) qRT-PCR showing relative levels of Arglu1 pre-mRNA normalized to Rp49 after alpha-amanitin treatment in Oregon R and clu mutant ovaries. Data are presented as mean values +/- SD from three biological replicates. *: p<0.05. Two-tailed t-test.

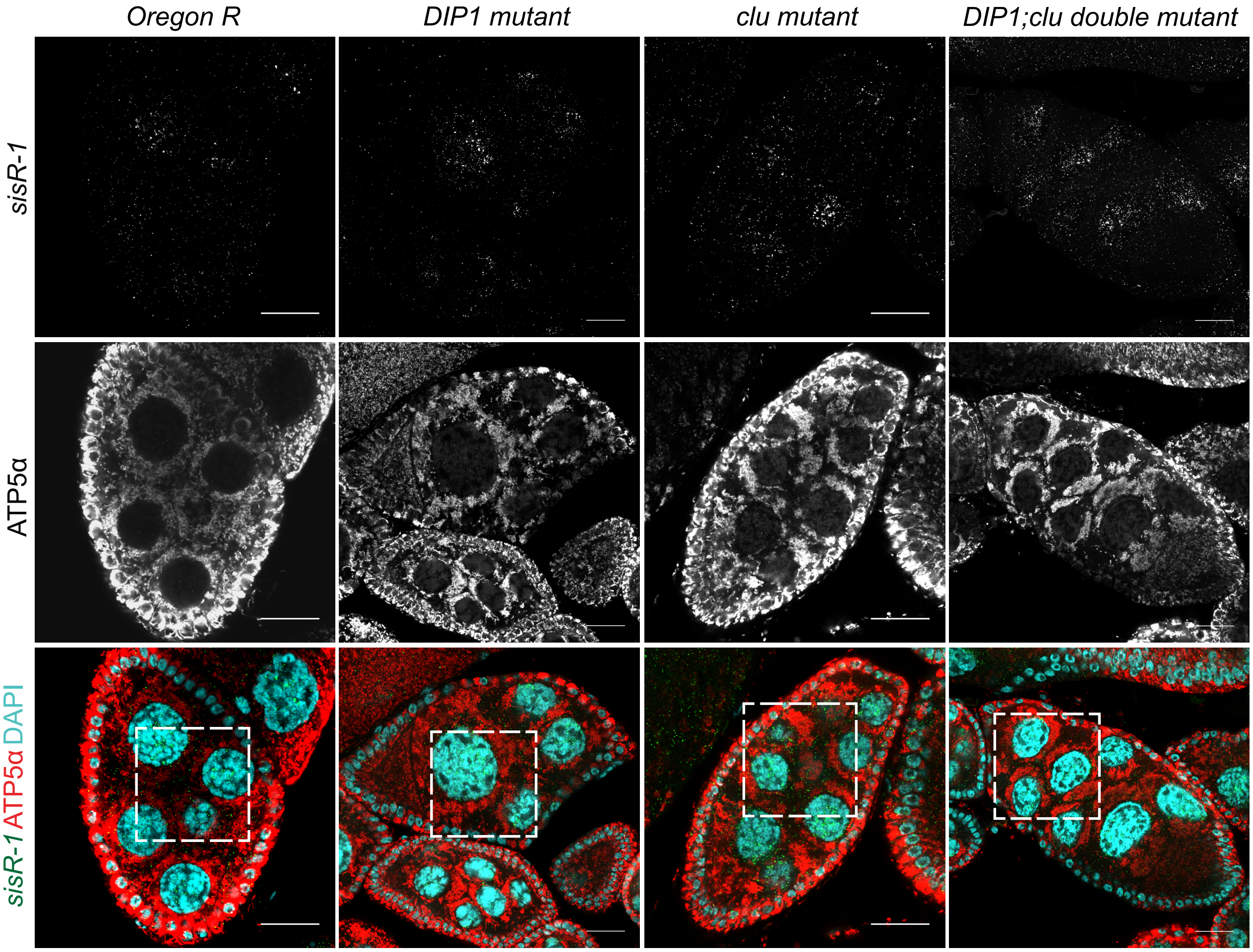

Fig S5. DIP1 and clu regulate sisR-1 and mitochondrial clumping. Confocal images of stage 8/9 egg chambers showing localization of sisR-1 (Green), ATP5α (Red) and DAPI (Blue) in fed Oregon R, DIP1 -/-, clu-/- and DIP1-/-;clu -/-. Scale bar = 20μm.

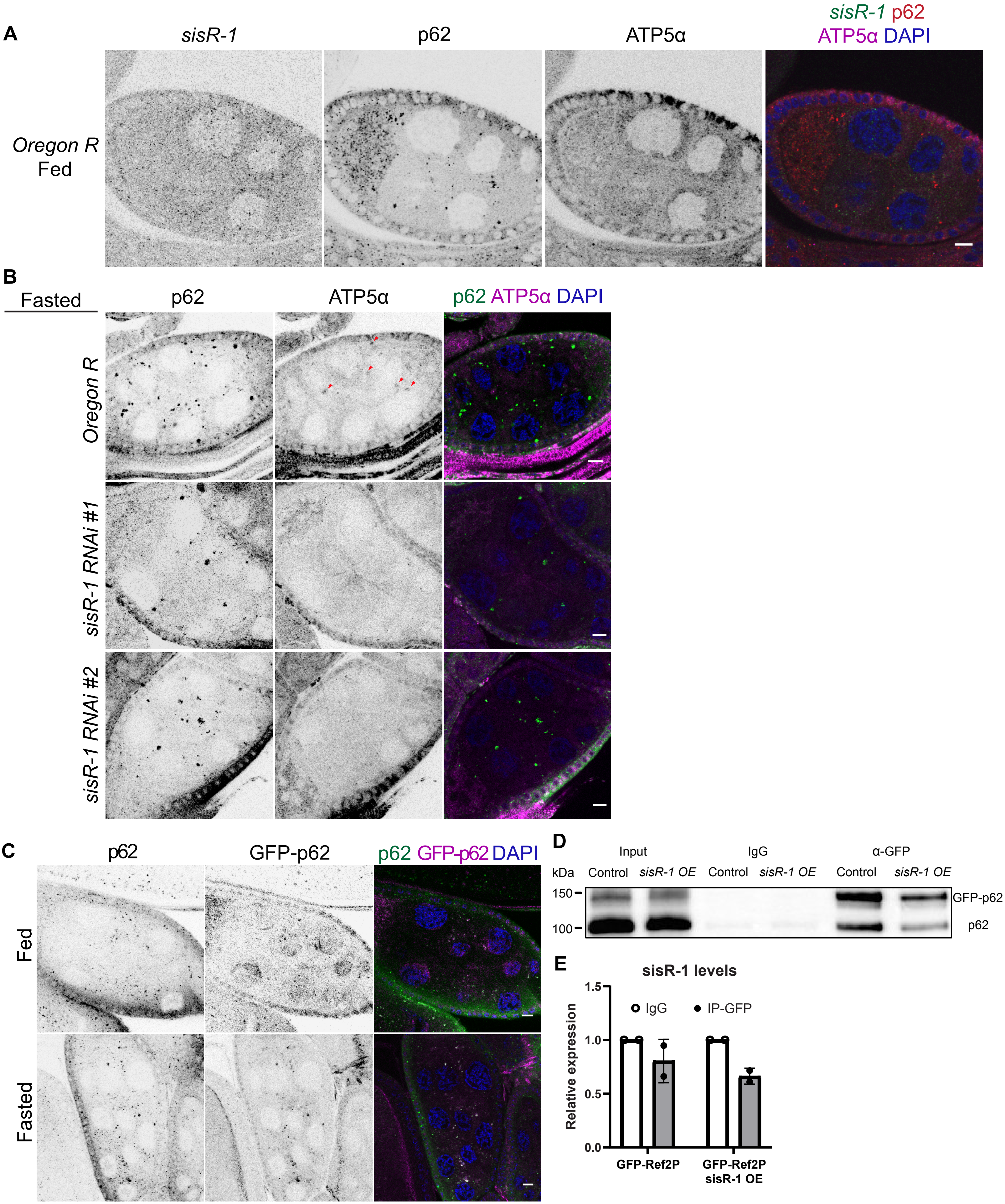

Fig S6. sisR-1 does not regulate p62 dimerization. (A) Confocal images of Oregon R fed with yeast paste

for 2 days. sisR-1 (Green), p62 (Red), ATP5α (Magenta) and DAPI (Blue). No colocalization is observed in fed Oregon R females. Scale bar = 10µm. (B) Confocal images of Oregon R, sisR-1 RNAi line 1 and 2 females fasted for 1 day. sisR-1 (Green), p62 (Red), ATP5α (Magenta) and DAPI (Blue). Arrowheads point to mitochondria clumps present in fasted Oregon R. Scale bar = 10µm. (C) Confocal images of nosP-GFP-p62 (on X) fed with yeast paste for 2 days and fasted post eclosure 1 day. p62 (Magenta), p62-GFP (Green) and DAPI (Blue). Scale bar = 10µm. (D) Western Blot showing immunoprecipitation of Oregon R and vasa-Gal4>sisR-1 OE ovaries fed with yeast paste for 2 days. Pull down of p62 using anti-GFP antibodies. Western blot is done using anti-p62 antibodies. Blot shows both GFP tagged and endogenous levels of p62. (E) qRT-PCR showing no enrichment of sisR-1 in GFP-p62 immunoprecipitates. Data are presented as mean values +/- SD from 2 biological replicates.
