# Supplementary material for: Nutrient-dependent regulation of a stable intron modulates germline mitochondrial quality control
Source: Nat Commun. 2024 Feb 10;15:1252. doi: 10.1038/s41467-024-45651-y (PMC10858910; doi:10.1038/s41467-024-45651-y)
Supplement: Supplementary file 3 — Reporting Summary [file 41467_2024_45651_MOESM3_ESM.pdf]

nature portfolio

Corresponding author(s): Jun Wei Pek

Last updated by author(s): 10 Jan 2024

Reporting Summary

Nature Portfolio wishes to improve the reproducibility of the work that we publish. This form provides structure for consistency and transparency in reporting. For further information on Nature Portfolio policies, see our Editorial Policies and the Editorial Policy Checklist.

Statistics

For all statistical analyses, confirm that the following items are present in the figure legend, table legend, main text, or Methods section.

n/a	Confirmed	
☐	☒	The exact sample size (*n*) for each experimental group/condition, given as a discrete number and unit of measurement
☐	☒	A statement on whether measurements were taken from distinct samples or whether the same sample was measured repeatedly
☐	☒	The statistical test(s) used AND whether they are one- or two-sided<br>*Only common tests should be described solely by name; describe more complex techniques in the Methods section.*
☒	☐	A description of all covariates tested
☒	☐	A description of any assumptions or corrections, such as tests of normality and adjustment for multiple comparisons
☐	☒	A full description of the statistical parameters including central tendency (e.g. means) or other basic estimates (e.g. regression coefficient) AND variation (e.g. standard deviation) or associated estimates of uncertainty (e.g. confidence intervals)
☐	☒	For null hypothesis testing, the test statistic (e.g. *F*, *t*, *r*) with confidence intervals, effect sizes, degrees of freedom and *P* value noted<br>*Give P values as exact values whenever suitable.*
☒	☐	For Bayesian analysis, information on the choice of priors and Markov chain Monte Carlo settings
☒	☐	For hierarchical and complex designs, identification of the appropriate level for tests and full reporting of outcomes
☒	☐	Estimates of effect sizes (e.g. Cohen's *d*, Pearson's *r*), indicating how they were calculated

Our web collection on statistics for biologists contains articles on many of the points above.

Software and code

Policy information about availability of computer code

Data collection	no software was used.
Data analysis	imageJ v1.47

For manuscripts utilizing custom algorithms or software that are central to the research but not yet described in published literature, software must be made available to editors and reviewers. We strongly encourage code deposition in a community repository (e.g. GitHub). See the Nature Portfolio guidelines for submitting code & software for further information.

Data

Policy information about availability of data

All manuscripts must include a data availability statement. This statement should provide the following information, where applicable:

- Accession codes, unique identifiers, or web links for publicly available datasets
- A description of any restrictions on data availability
- For clinical datasets or third party data, please ensure that the statement adheres to our policy

The data that support the findings of this study are available in the article, Supplementary Information and from the corresponding author upon reasonable request. Source data are provided with this paper.

Research involving human participants, their data, or biological material

Policy information about studies with human participants or human data. See also policy information about sex, gender (identity/presentation), and sexual orientation and race, ethnicity and racism.

Reporting on sex and gender	na
Reporting on race, ethnicity, or other socially relevant groupings	na
Population characteristics	na
Recruitment	na
Ethics oversight	na

Note that full information on the approval of the study protocol must also be provided in the manuscript.

Field-specific reporting

Please select the one below that is the best fit for your research. If you are not sure, read the appropriate sections before making your selection.

☒ Life sciences ☐ Behavioural & social sciences ☐ Ecological, evolutionary & environmental sciences

For a reference copy of the document with all sections, see nature.com/documents/nr-reporting-summary-flat.pdf

Life sciences study design

All studies must disclose on these points even when the disclosure is negative.

Sample size	Samples sizes were not pre-determined prior to the experiments. For qPCR, standard practice is to have n=3. For others, n>8 is used to display normal distribution.
Data exclusions	no data were excluded.
Replication	all attempts at replications were successful. For qPCR, 3 technical replicates and 3 biological replicates were performed. For other experiments, a minimum of 3 biological replicates were performed.
Randomization	Samples were randomly selected for treatment or analyses without any preconceived bias.
Blinding	Investigators were not blinded. Due to manpower constraints, the investigators experiments were performed and analyzed by same person.

Reporting for specific materials, systems and methods

We require information from authors about some types of materials, experimental systems and methods used in many studies. Here, indicate whether each material, system or method listed is relevant to your study. If you are not sure if a list item applies to your research, read the appropriate section before selecting a response.

Materials & experimental systems

n/a	Involved in the study	
☐	☒	Antibodies
☒	☐	Eukaryotic cell lines
☒	☐	Palaeontology and archaeology
☐	☒	Animals and other organisms
☒	☐	Clinical data
☒	☐	Dual use research of concern
☒	☐	Plants

Methods

n/a	Involved in the study	
☒	☐	ChIP-seq
☒	☐	Flow cytometry
☒	☐	MRI-based neuroimaging

Antibodies

Antibodies used	For western blots, antibodies used were rabbit anti-DIP1 (1:5,000, Pek lab), rabbit anti-p62 (1:1,000, gift from Shoichiro Kurata and Tamaki Yano), mouse anti-porin (1:200, abcam 14734), mouse anti-lamin (1:1,000, DSHB ADL67.10-c), mouse anti-GFP (1:2,000, Invitrogen 3E6 A-11120), mouse anti-ATP5a (1:1000, Abcam 15H4C4), mouse anti-beta-Tubulin (1:1000, DSHB E7) and mouse anti-

	Actin (1:100, DSHB JLA20).<br>For immunostaining, antibodies used were as follows: mouse anti-ATP5A (1:500, Abcam 14748), rabbit anti-p62 (1:1000, gift from Shoichiro Kurata and Tamaki Yano), rabbit anti-DIP1 (1:300. Pek lab).
Validation	DIP1: Wong, J. T. et al. DIP1 modulates stem cell homeostasis in Drosophila through regulation of sisR-1. Nature communications 8, 759, doi:10.1038/s41467-017-00684-4 (2017).<br>p62: Nagai, H., Tatara, H., Tanaka-Furuhashi, K., Kurata, S. & Yano, T. Homeostatic Regulation of ROS-Triggered Hippo-Yki Pathway via Autophagic Clearance of Ref(2)P/p62 in the Drosophila Intestine. Developmental cell 56, 81-94 e10, doi:10.1016/j.devcel.2020.12.007 (2021).<br>Porin: https://www.abcam.com/en-sg/products/primary-antibodies/vdac1-porin-vdac3-antibody-20b12af2-ab14734<br>Lamin: https://dshb.biology.uiowa.edu/ADL67-10<br>GFP: https://www.thermofisher.com/antibody/product/GFP-Antibody-clone-3E6-Monoclonal/A-11120<br>ATP5a: https://www.abcam.com/en-sg/products/primary-antibodies/atp5a-antibody-15h4c4-mitochondrial-marker-ab14748<br>Tubulin: https://dshb.biology.uiowa.edu/E7_2

Animals and other research organisms

Policy information about studies involving animals; ARRIVE guidelines recommended for reporting animal research, and Sex and Gender in Research

Laboratory animals	The following fly strains were used in this study: y w and Oregon R (used as control unless otherwise stated), vasa-Gal4/CyO, TOR RNAi[HMS00904] (Bloomington #33951), sisR-1 RNAi #1 and #2, sisR-1 OE, clu[d08713/CyO] and clu[CA06604] (gifts from Rachel Cox), DIP1 (Bloomington #15577), GFP-p62 (gift from Bhupendra Shravage), UAS-park41, UAS-porin/VDAC1 WT, UAS-porin/VDAC1 K273R (mono), UAS-porin/VDAC1 poly-KR (poly). To knockdown sisR-1, UASp-sisR-1 RNAi were crossed with daughterless (da)-Gal4 as described previously. All animals were ~2-7 days old unless those used in aging experiments were 4 and 12 days old.
Wild animals	No wild animals were used in the study.
Reporting on sex	only females were used as we study oogenesis.
Field-collected samples	No field collected samples were used in the study.
Ethics oversight	Ethics approval not required for Drosophila work.

Note that full information on the approval of the study protocol must also be provided in the manuscript.

Plants

Seed stocks	na
Novel plant genotypes	na
Authentication	na
