# Supplementary material for: Nutrient-dependent regulation of a stable intron modulates germline mitochondrial quality control
Source: Nat Commun. 2024 Feb 10;15:1252. doi: 10.1038/s41467-024-45651-y (PMC10858910; doi:10.1038/s41467-024-45651-y)
Supplement: Supplementary file 4 — Source data [file 41467_2024_45651_MOESM4_ESM.zip › Source Data/Source Data.pdf]

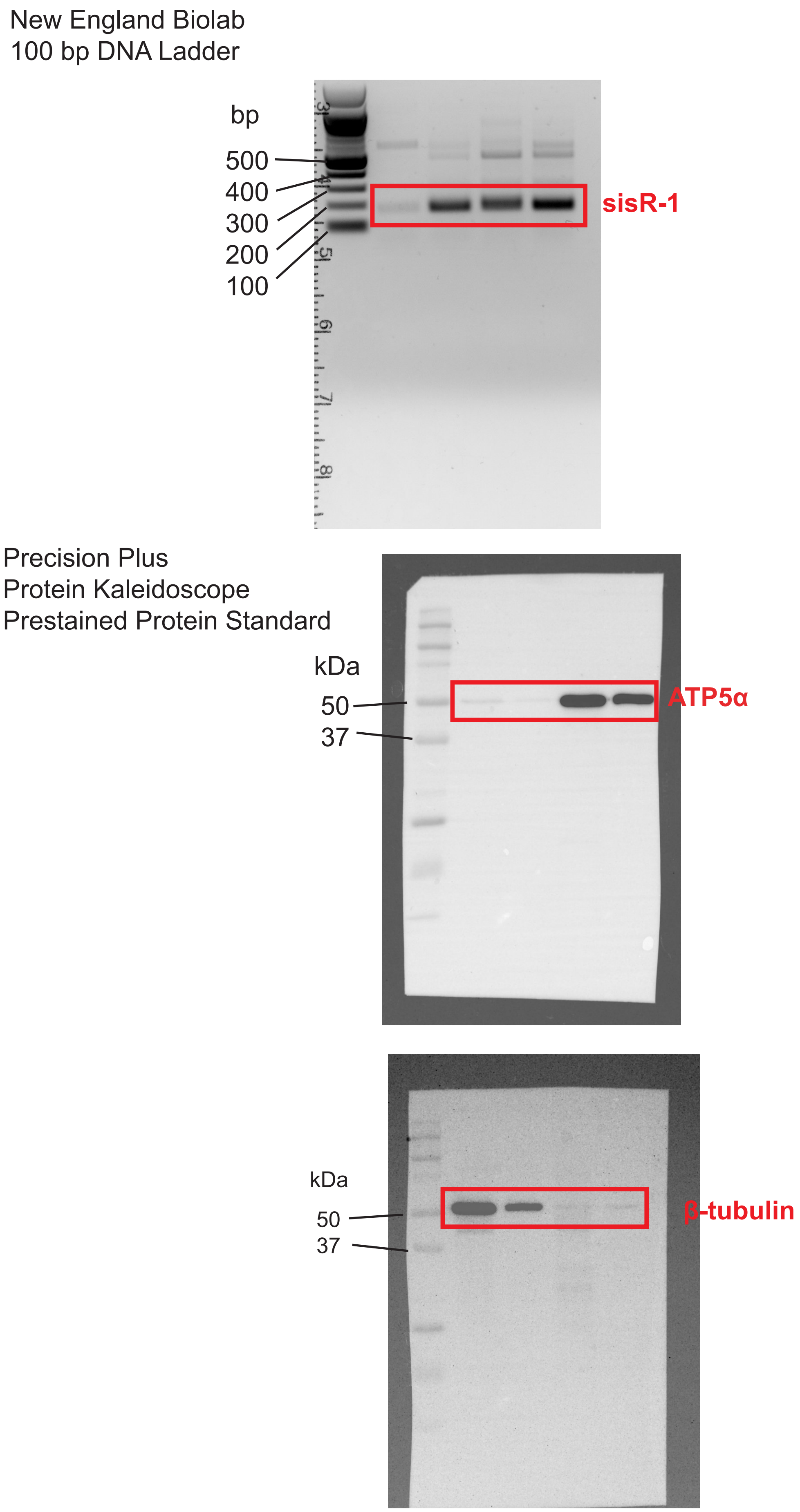

Figure 1G original image

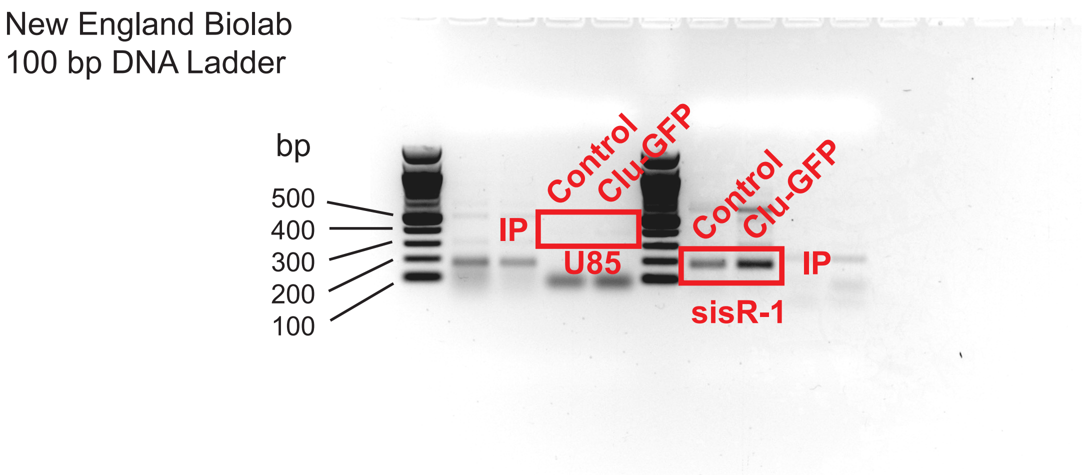

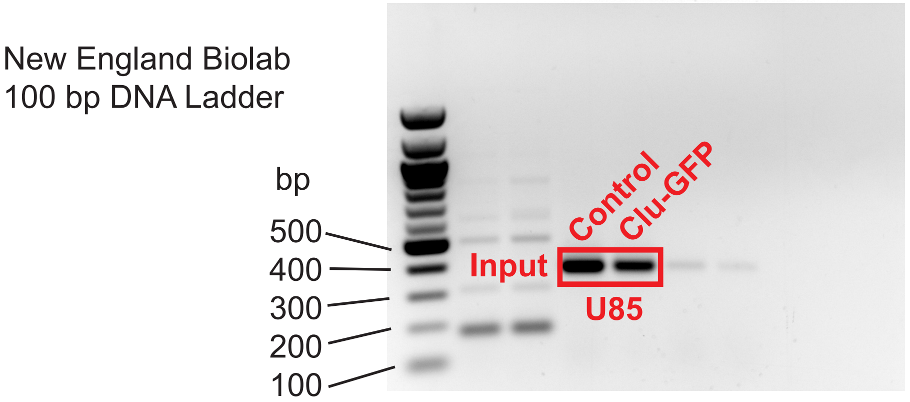

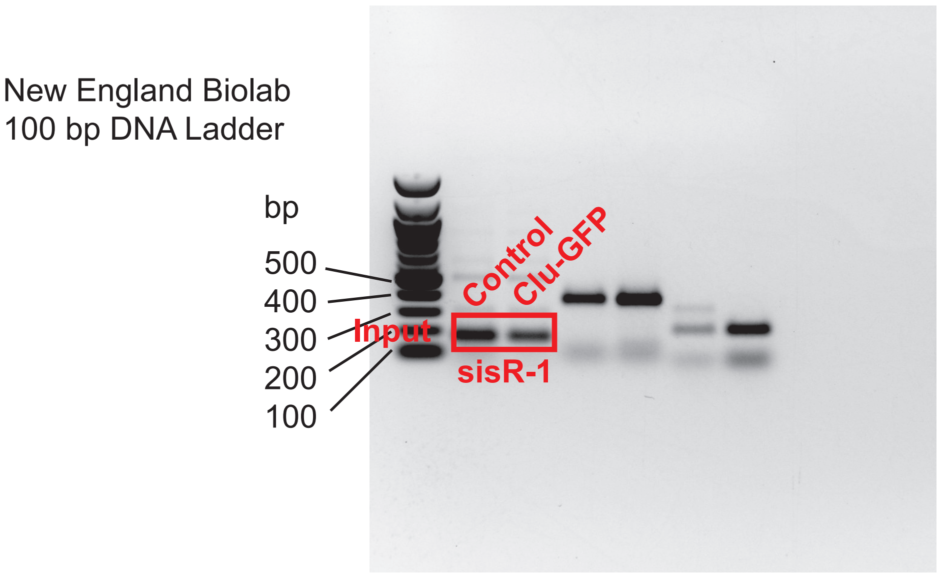

Figure 2F original gel image

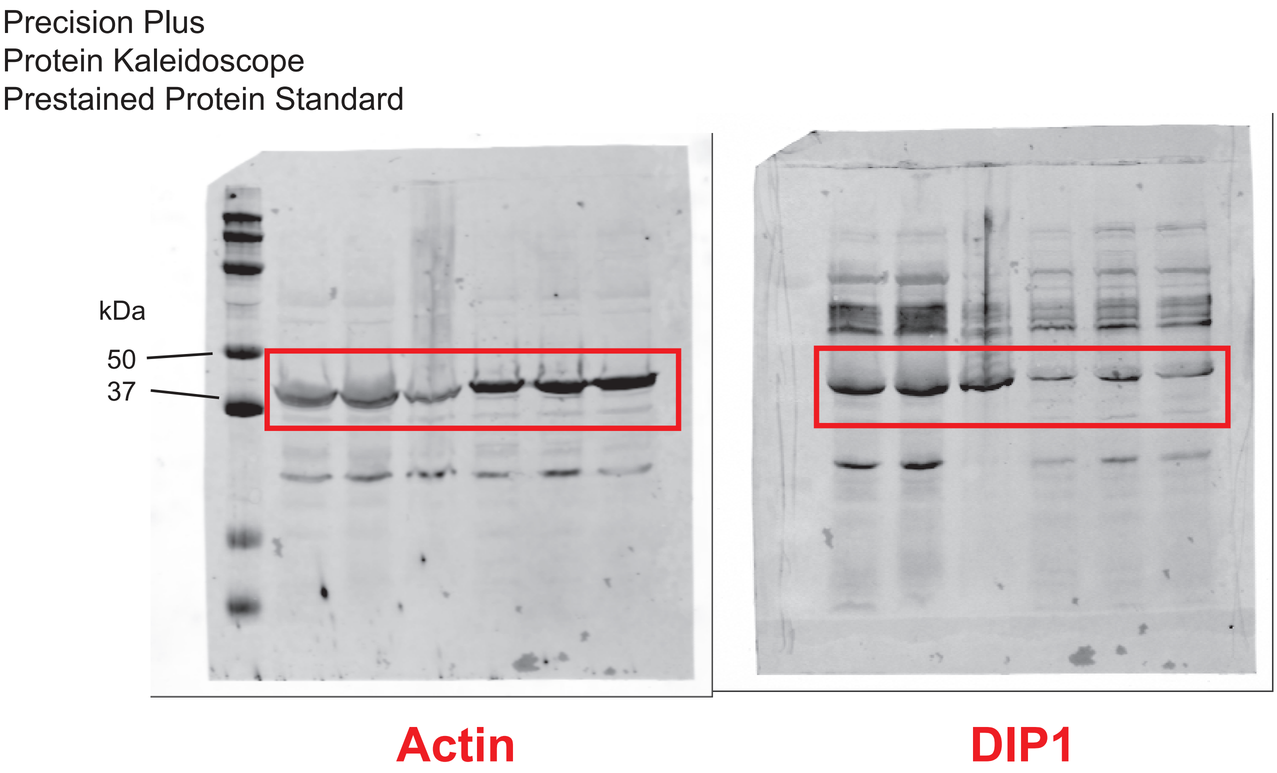

Figure 3B original image

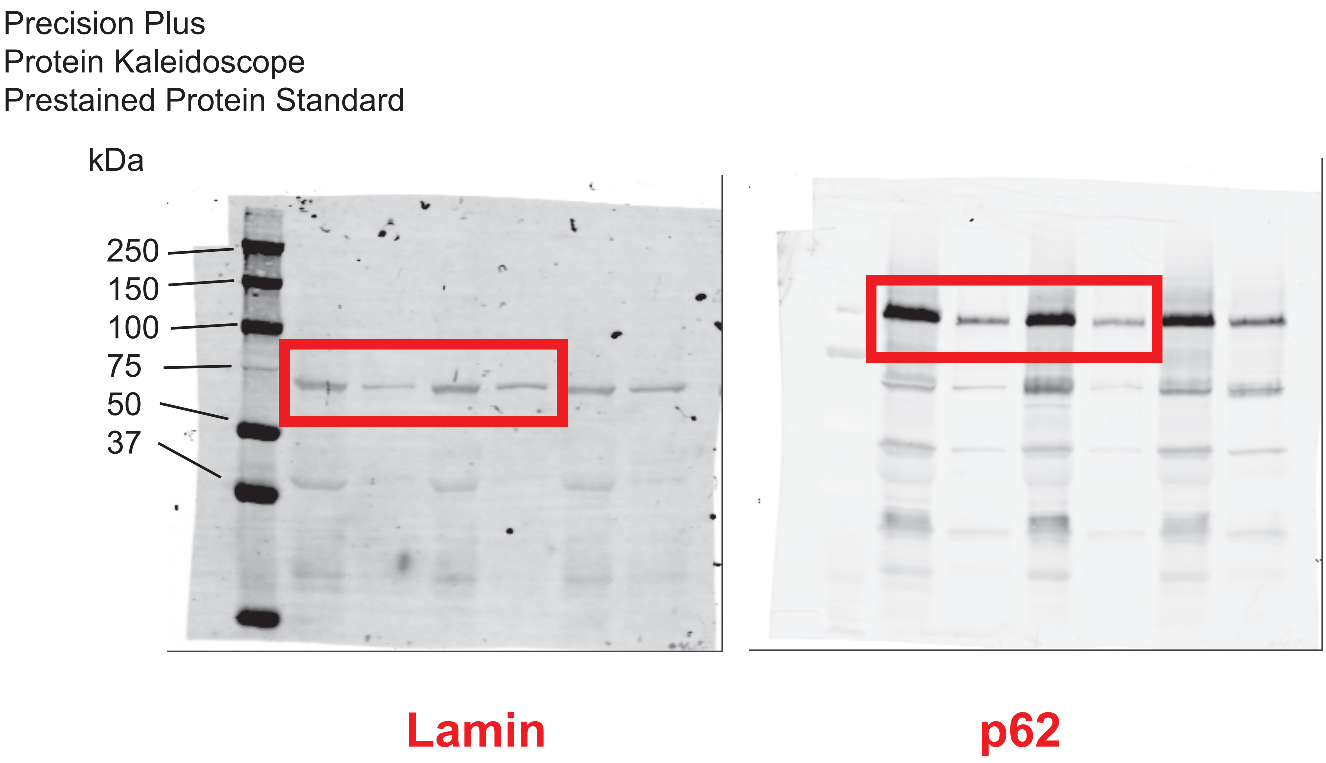

Figure 4D original image

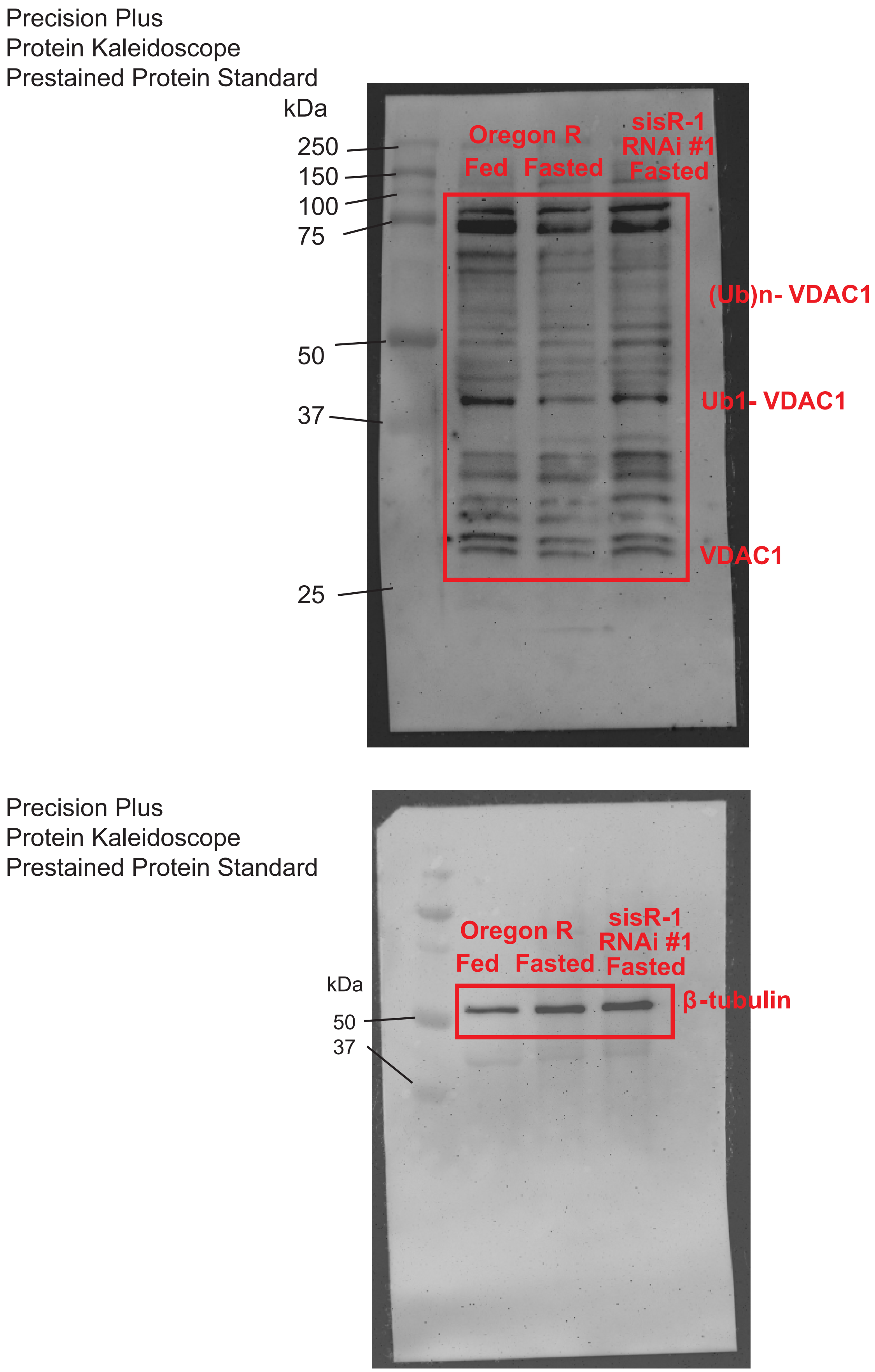

Figure 4H original image

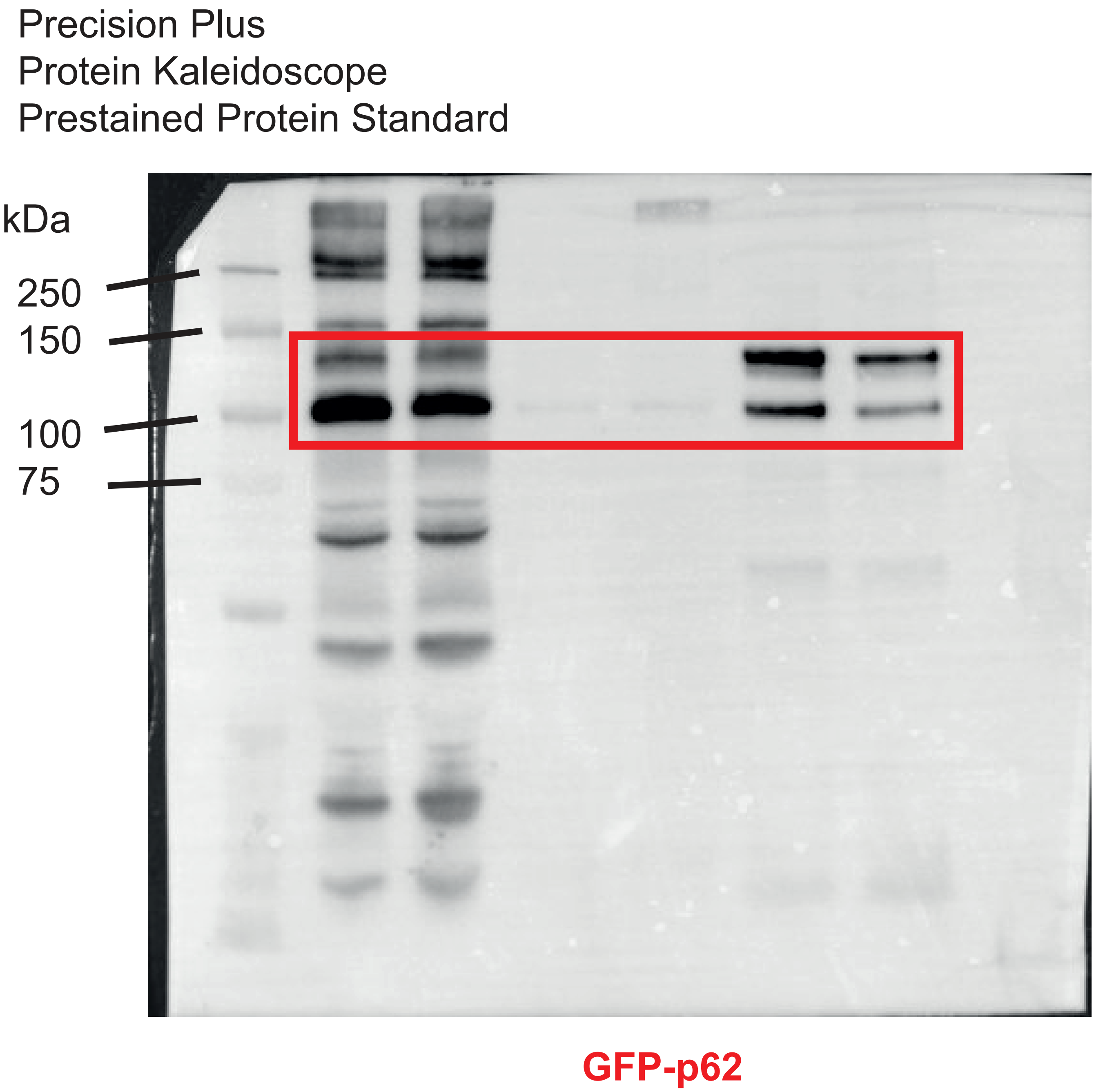

Figure S6D original image
